# A Polysiloxane Delivery Vehicle of Cyclic *N*-Halamine for Biocidal Coating of Cellulose in Supercritical CO_2_

**DOI:** 10.3390/polym14235080

**Published:** 2022-11-23

**Authors:** Leixuan Li, Yan Xin, Fengze Wu, Xiangrong Lyu, Qiyuan Yao, Xiaoting Yin, Qiang Zhang, Wenjuan Shan, Yong Chen, Qiuxia Han

**Affiliations:** 1 Department of Applied Chemistry, College of Chemical and Biological Engineering, Shandong University of Science and Technology, Qingdao 266590, China; 2 Analytical and Testing Center, School of Materials Science and Engineering, Shandong University of Science and Technology, Qingdao 266590, China; 3 College of Chemistry and Chemical Engineering, Liaoning Normal University, 850 Huanghe Road, Dalian 116029, China; 4 Department of Biological Engineering, College of Chemical and Biological Engineering, Shandong University of Science and Technology, Qingdao 266590, China

**Keywords:** cyclic imide *N*-halamines, polysiloxane, delivery vehicle, antibacterial surface, cellulose, scCO_2_ interpenetration

## Abstract

Cyclic *N*-halamines are highly antimicrobial, very stable, and not susceptible to bacterial resistance. A polysiloxane delivery vehicle was synthesized to deliver cyclic imide *N*-halamine onto cellulose via a benign and universal procedure that does not require a harmful solvent or chemical bonding. In brief, Knoevenagel condensation between barbituric acid and 4-hydroxybenzaldehyde furnished 5-(4-hydroxybenzylidene)pyrimidine-2,4,6-trione, whose phenolic O−H was subsequently reacted with the Si−H of poly(methylhydrosiloxane) (PMHS) via silane alcoholysis. The product of silane alcoholysis was interpenetrated into cellulose in supercritical CO_2_ (scCO_2_) at 50 °C, to form a continuous modification layer. The thickness of the modification layer positively correlated with interpenetration pressure in the experimental range of 10 to 28 MPa and reached a maximum value of 76.5 nm, which demonstrates the ability for tunable delivery, to control the loading of the imide N−H bond originating from barbituric acid unit. The imide N−H bonds on cellulose with the thickest modifier were then chlorinated into N−Cl counterparts using *tert*-butyl hypochlorite, to exert a powerful biocidability, providing ~7 log reductions of both *S. aureus* and *E. coli* in 20 min. The stability and rechargeability of the biocidability were both very promising, suggesting that the polysiloxane modifier has a satisfactory chemical structure and interlocks firmly with cellulose via scCO_2_ interpenetration.

## 1. Introduction

Antibacterial materials can prevent bacterial infection and hence are very useful in hygiene and daily life [1,2]. It is of great importance to modify materials for durable biocidability, with a powerful biocide and a facile and green procedure [3]. Compared with other commonly used polymers, such as polyolefins, cellulose is more susceptible to bacterial attachment and proliferation, due to its surface hydrophicility, which origates from the hydroxy groups [4]. It is therefore particularly significant to use cellulose as a reprensatitive to evaluate the performace of biocides and modification procedures.

Many biocidal functionalities such as *N*-halamines (*N*-chloramines and *N*-bromamines) [5,6,7], antibiotics [8,9], quaternary ammonia salts [10,11,12], reactive oxygen species [13,14], and heavy-metal ions [15,16,17,18] have been incorporated onto cellulose to give an antibacterial ability. *N*-halamines, produced by halogenation of imide/amide/amine N−H to N−X (X=Cl, Br), have many advantages, since they are highly powerful, rechargeable, broad-spectrum, and not susceptible to bacterial resistance. The positively charged halogen atoms in *N*-halamines are strong oxidizers that can the destroy enzymatic and/or metabolic activities of microorganisms [19]. Among the three types of *N*-halamine, the imide type is the most effective, since it has the highest dissociation constant, allowing the fastest release of oxidative halogen ions [20]. In addition, a cyclic *N*-halamine is more stable than an acyclic one [21,22,23]. Based on these findings, a cyclic imide *N*-halamine is expected to be an ideal candidate for biocidal applications.

Barbituric acid is a precursor of *N*-halamine, since it is cyclic and has two imide N−H bonds for halogen binding. Small biocidal compounds including barbituric acid-based *N*-halamines, however, cannot tightly attach to a surface since they have limited binding sites with substrates. The leaching of small molecules causes a loss of biocidability of the substrate, pollution of the environment, and harmful interpenetration into human skin [24]. Therefore, it is preferable to use the polymeric form of *N*-halamine to modify a substrate.

Barbituric acid needs functional groups to form polymers. For instance, a carbon–carbon double bond was introduced at C5 of barbituric acid for radical polymerization [25,26]. The functionalization at C5 using barbituric acid as a starting material is unspecific, since it can also occur at the two imide nitrogen atoms. To avoid this complication, the traditional solution was to modify the central carbon of diethyl malonate for the desired functionality by nucleophilic substitution. The resultant product was subsequently reacted with urea through condensation. However, this procedure has its own drawbacks, including a low yield, hydrolysis of malonate, decarbethoxylation, and thermal degradation of urea [27].

Knoevenagel condensation between barbituric acid and aromatic aldehydes provides a solution to the abovementioned problems, allowing a specific mono-functionalization at C5 of barbituric acid. In addition, this condensation is performed in water and does not need a catalyst, and therefore is was chosen for this study. Herein, the used aromatic aldehyde is 4-hydroxybenzaldehyde, to introduce a phenolic OH group at C5 of barbituric acid.

Biocidal functionality has been given to cellulose using a variety of procedures. To the best of our knowledge, the only physical procedure that has been applied to cellulose for repeated usage is layer-by-layer assembly [4,28]. However, this method works better on a highly charged surface, which cellulose is not. Moreover, the anionic species in the polyelectrolyte multilayer repels negatively-charged bacteria, which lowers the contact-killing efficacy. Cellulose has hydroxy bonds that can be used to chemically connect biocidal groups. However, the areal density of the hydroxy bond is low, such that it is insufficient to generate desirable biocidability if each hydroxy bond only connects with one biocidal group. There are two common methods to form biocidal polymers, each of whose segment has one or more biocidal groups on cellulose, to compensate for the low hydroxy density. One is the formation of radicals on the surface for “grafting from” polymerization of biocidal monomers [29,30,31,32]. The other is the use of silanes as biocidal carriers. Silanes are hydrolyzed to convert alkoxysilyl groups to silanol ones. The silanol groups of different hydrolyzates condense, not only with one another, but also with hydroxy groups of cellulose, furnishing a polysiloxane layer [33,34,35].

Herein, a physical and chemical combined procedure named scCO_2_ interpenetration is used to modify cellulose. This procedure does not require reactivity or a polluting solvent and, hence, is universal and benign. The principle of this procedure is that a soluble modifier penetrates into a swollen substrate in the working solvent of scCO_2_, to obtain a new interfacial property. Besides low-molecular-weight compounds, only a very few polymers, including polysiloxanes, fluorinated macromolecules, and poly(ether-carbonate) copolymers are soluble, since scCO_2_ has a very poor solvent strength [36,37]. However, almost any polymeric substrate will swell greatly in scCO_2_, due to its a superior interpenetration ability. Therefore, a modifier can be a small monomer that polymerizes to long chains after interpenetration, to entangle with those of the substrate or a CO_2_-soluble polymer that does not require post-polymerization. In both cases, a polymeric modifier interlocks firmly with the substrate to provide a new interfacial property. ScCO_2_ interpenetration is especially useful for modification of substrates such as polyolefins and cellulose that do not swell in common liquid solvents and do not have the required reactivity. Among the few CO_2_-soluble polymers, polysiloxanes are usually chosen as a delivery vehicle to convey the desired functionalities such as biocidability onto substrates, since they are industrially available, nonhazardous, chemically stable, and resistant to a wide range of temperatures. The synthesis of such a delivery vehicle involves the incorporation of a functional group to a backbone composed of a –Si–O– repeat unit.

**Scheme 1:**
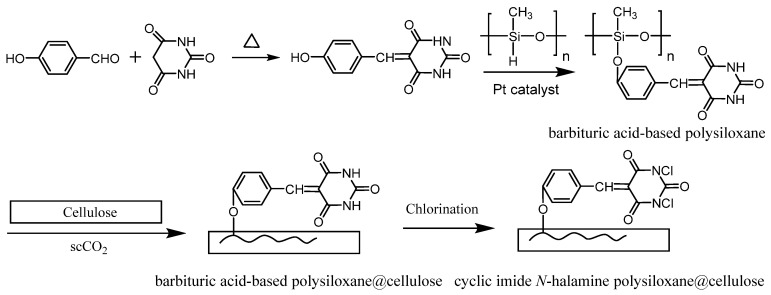
Fabrication of cyclic imide *N*-halamine polysiloxane@cellulose.

Based on the above discussion, using scCO_2_ interpenetration to insert a polysiloxane delivery vehicle of cyclic imide *N*-halamine groups is hypothesized to be a versatile and friendly solution to forming a powerful and durable biocidability on cellulose. To verify this assumption, a polysiloxane with barbituric acid pendants was synthesized and interpenetrated into cellulose in scCO_2_, to form a stable interlocking layer. Subsequent chlorination of N−H bonds of barbituric acid furnished a polysiloxane modifier with cyclic imide *N*-halamine sites. Briefly, Knoevenagel condensation between barbituric acid and 4-hydroxybenzaldehyde furnished 5-(4-hydroxybenzylidene)pyrimidine-2,4,6-trione. The phenolic hydroxy group of 5-(4-hydroxybenzylidene)pyrimidine-2,4,6-trione was reacted with of Si−H of PMHS via silane alcoholysis, to produce a biocidal polysiloxane precursor (referred to as barbituric acid-based polysiloxane). The barbituric acid-based polysiloxane was interpenetrated into cellulose in scCO_2_ at 50 °C, the temperature at which polysiloxanes are most soluble [36], to furnish a sample named barbituric acid-based polysiloxane@cellulose. Since the pressure of scCO_2_ is a crucial parameter affecting the solubility and hence the thickness of barbituric acid-based polysiloxane, the relationship between interpenetration pressure and thickness of barbituric acid-based polysiloxane was studied. The results showed that the thickness of the barbituric acid-based polysiloxane was able to be conveniently adjusted by variation of the interpenetration pressure, to deliver different amounts of the *N*-halamine precursor, imide N−H from barbituric acid. Cellulose with the maximum loading of barbituric acid-based polysiloxane was chlorinated to convert N−H bonds using *tert*-butyl hypochlorite, to create a sample with cyclic imide *N*-halamine sites on the surface, which is hereafter called cyclic imide *N*-halamine polysiloxane@cellulose. The overall procedure is shown in Scheme 1.

Antibacterial and stability tests showed that the resultant cyclic imide *N*-halamine polysiloxane@cellulose possessed a super, stable, and rechargeable biocidability, as expected, confirming the benefits of a cyclic imide *N*-halamine, polysiloxane delivery vehicle and scCO_2_ interpenetration.

## 2. Materials and Methods

### 2.1. Materials

Pt catalyst, Platinum(0)-1,3-divinyl-1,1,3,3-tetramethyldisiloxane, was purchased from Shanghai Silicon Mountain Macromolecular Materials Co., Ltd. (Shanghai, China). Cellulose swatch was supplied by Weifang Jinda Textile Co., Ltd. (Weifang, China). Poly(methylhydrosiloxane) (PMHS) (Mn: 3.9 × 10^3^, Mw/Mn = 3.0) was purchased from Bayee Chemical Co., Ltd. (Zibo, China). Barbituric acid, anhydrous calcium chloride and 4-hydroxybenzaldehyde were supplied by Shanghai Aladdin Bio-Chem Technology Co., Ltd. (Shanghai, China). Sodium thiosulfate, sodium hypochlorite (10 wt%) and phosphate buffered saline (PBS) were obtained from China National Pharmaceutical Group Corporation (Beijing, China). Glacial acetic acid was acquired from J&K Scientific Ltd. (Beijing, China). *Tert*-butyl alcohol was purchased from Beijing Tianyukanghong Chemical Technology Co., Ltd. (Beijing, China). *Escherichia coli* and *Staphylococcus aureus* were purchased from Guangdong Institute of Microbiology (Guangzhou, China). Carbon dioxide (99.99%) was supplied by Qingdao Sanyinzi Gas Technology Co., Ltd. (Qingdao, China). All other chemicals were supplied by Qingdao Xiangrong Technology Co. Ltd. (Qingdao, China).

### 2.2. Instrumentation

Fourier transform infrared spectroscopy (FTIR) spectra were acquired with a Nicolet Magna IR-560 FTIR spectrometer(Madison, WI, USA), using KBr pellet method at a resolution of 0.5 cm^−1^ in the wavelength range of 400 to 4000 cm^−1^.

The types and valences of elements on cellulose were measured with a Thermo Scientific Escalab 250Xi X-ray photoelectron spectrometer (XPS) (Waltham, MA, USA) equipped with a Al Ka radiation source and a low energy electron flood gun. The test pressure and takeoff angle were maintained at 1 × 10^−6^ Pa and 45°, respectively, during spectrum acquisitions. Survey spectra were collected at an analyzer pass energy of 100 eV and a resolution of 1 eV, while high resolution spectra were collected at an analyzer pass energy of 23.5 eV and a resolution of 0.05 eV. Elemental binding energies were referenced to the aliphatic C_1s_ at 284.7 eV.

Pristine cellulose and cyclic imide *N*-halamine polysiloxane@cellulose were first coated with platinum under vacuum with a Cressington 108 auto sputter coater and then observed with a FEI Nano SEM450 scanning electron microscope (Waltham, MA, USA) that was equipped with a secondary electron detector. SEM images were collected at an accelerating voltage of 10 kV and a chamber pressure of 1 × 10^−4^ Pa.

The density of barbituric acid-based polysiloxane was measured using a density balance (FA2004J) from Shanghai Pingxuan Science Instrument Co., Ltd. (Shanghai, China).

### 2.3. Synthesis of Barbituric Acid-Based Polysiloxane That Bears Cyclic Imide N−H Sites

The synthesis of barbituric acid-based polysiloxane involves two steps, as shown in Scheme 1. First, 2.56 g (0.02 mol) of barbituric acid and 2.44 g of (0.02 mol) 4-hydroxybenzaldehyde were dissolved in 50 mL water in a flask. The solution was stirred at 70 °C for 6 h to complete the Knoevenagel condensation and furnish 5-(4-hydroxybenzylidene)pyrimidine-2,4,6-trione, which was purified by recrystallization from water.

The phenolic O−H of 5-(4-hydroxybenzylidene)pyrimidine-2,4,6-trione was subsequently reacted with Si−H of PMHS. 2.32 g (0.01 mol) of 5-(4-hydroxybenzylidene)pyrimidine-2,4,6-trione, two drops of Pt catalyst, and 0.70 g (0.012 mol of Si−H) of PMHS were dissolved in 60 mL of dry tetrahydrofuran (THF) in a 150 mL three-necked round-bottom flask. The solution was refluxed for 6 h under nitrogen to finish the silane alcoholysis. The solvent was removed with a rotary evaporator at reduced pressure, to obtain viscous barbituric acid-based polysiloxane.

### 2.4. Interpenetration of Cellulose with Barbituric Acid-Based Polysiloxane in scCO_2_

A 100 mL high-pressure stainless-steel cell was separated into an upper and a lower chamber using a removable perforated steel slice. A 5.0 × 5.0 cm^2^ cellulose swatch (about 0.26 g) was placed in the upper chamber and a glass vial containing 0.4 g of barbituric acid-based polysiloxane was placed in the lower one, to avoid direct contact. The cell was then purged with CO_2_, heated to 50 °C and charged with CO_2_ to a desired pressure with a syringe pump. The cell was maintained under these conditions overnight, to complete one interpenetration process. The pressurization and depressurization periods were about 3 and 5 min, respectively. An average of three measurements was reported for each interpenetration condition. A temperature of 50 °C was selected as polysiloxanes have maximum solubility at this temperature [36]. An overnight interpenetration period was chosen since a longer time did not cause a measurable change of the loading of barbituric acid-based polysiloxane on cellulose. It is known that the solubility of polysiloxanes increases with the increase of pressure [38], indicating a facile way to form an interpenetration layer with adjustable thickness, to achieve an improved delivery ability. Herein, the thickness of barbituric acid-based polysiloxane was controlled by varying the interpenetration pressure between 8 and 28 MPa, which in turn decided the amount of cyclic imide *N*-halamine that was delivered to the final cellulose surface.

The thickness of the barbituric acid-based polysiloxane modifier at a certain pressure can be estimated based on the weight change before and after the interpenetration process using the following Equation (1) [33]:

(1) $$h=\frac{d\left(W_{1}-W_{0}\right)\rho_{\mathrm{cellulose}}}{4W_{0}\rho_{\mathrm{polysiloxane}}}$$

where h is the layer thickness of barbituric acid-based polysiloxane, d is the average diameter of the original cellulose fiber (11.8 μm), $W_{0}$ is the weight of the original fibers used for interpenetration, $W_{1}$ is the weight of fibers after interpenetrated with barbituric acid-based polysiloxane, $\rho_{\mathrm{cellulose}}$ is the density of cellulose (1.54 g/cm^3^), and $\rho_{\mathrm{polysiloxane}}$ is the density of barbituric acid-based polysiloxane (0.99 g/cm^3^). Each sample was tested in triplicate.

The cellulose with the thickest interpenetration layer was chosen for further modifications and characterizations including chlorination, biocidal assessments, and stability tests.

### 2.5. Chlorination of Cyclic Imide N−H Sites of Barbituric Acid-Based Polysiloxane

For chlorination of polymers including barbituric acid-based polysiloxane, *tert*-butyl hypochlorite performs better than inorganic sodium hypochlorite, due to the “like dissolves like” principle and hence was used in this study. *Tert*-butyl hypochlorite was prepared using sodium hypochlorite, glacial acetic acid, and *tert*-butyl alcohol, according to a reported procedure based on the following reaction (2) [39].

(CH_3_)_3_COH + CH3COOH + NaClO = (CH_3_)_3_COCl + CH_3_COONa +H_2_O (2)

Cellulose fabrics (5.0 × 5.0 cm^2^) with the thickest interpenetration layer were then immersed in excessive *tert*-butyl hypochlorite in a flask at room temperature overnight to chlorinate the cyclic imide N−H sites of barbituric acid-based polysiloxane to their N−Cl counterparts. After that, excessive *tert*-butyl hypochlorite was vacuum dried to obtain cyclic imide *N*-halamine polysiloxane@cellulose, as shown in Scheme 1.

### 2.6. Titration of Biocidal Chlorine

The loading of biocidal chlorine on cyclic imide *N*-halamine polysiloxane@cellulose was assessed using iodimetric/thiosulfate titration for correlation with a biocidability that is concentration dependent. Before titration, the cyclic imide *N*-halamine polysiloxane@cellulose fabrics were initially washed with DI water, in order to remove free chlorines and then dried in the air. Pristine cellulose fibers were subjected to the same treatments and served as controls. Each sample was tested in triplicate. The loading of biocidal chlorine on cellulose was calculated using the following Equation (3) [40]:

(3) $$m_{{Cl}^{+}}\%=\frac{35.5N\times \left(V_{Cl^{+}}-V_{0}\right)\times 100}{2W}$$

where $m_{Cl^{+}}\%$ is the weight percentage of biocidal chlorine on cyclic imide *N*-halamine polysiloxane@cellulose fibers, *N* is the concentration (mol/L) of Na_2_S_2_O_3_ solution for titration, *V_Cl_^+^* and *V*_0_ represent the volumes (L) of Na_2_S_2_O_3_ solution used for titrations of cyclic imide *N*-halamine polysiloxane@cellulose fibers and pristine cellulose fibers, respectively, and *W* is the weight (g) of cellulose fibers in titrations.

### 2.7. Antibacterial Kinetics of Cyclic Imide N-Halamine Polysiloxane@Cellulose Fabrics

The biocidal kinetics of cyclic imide *N*-halamine polysiloxane@cellulose fabrics were measured by challenging with Gram-negative *E. coli* and Gram-positive *S. aureus* in a “sandwich test”, using pristine ones as negative controls [41]. *E. coli* and *S. aureus* were first cultured in Luria–Bertani (LB) and Tryptic Soy (TS) broths, respectively, at 37 °C for 24 h. The bacteria were then separated with the aid of centrifugation, washed several times with sterilized PBS solution, and finally adjusted to a concentration of ~10^8^ CFU/mL with PBS. Then, 25 μL of the suspension was sandwiched between two pieces of identical swatches of 2.54 cm^2^ each. A sterilized weight was placed on the top to ensure good contact of the swatches and bacterial suspension. The swatches of different contact periods were soaked in 10 mL of sterile Na_2_S_2_O_3_ (0.05 wt%) in a tube, to neutralize biocidal chlorines and vortexed for 2 min, to remove the surface-attached bacteria. Next, 100 μL of each serial dilution of a vortexed solution was placed on an agar plate at 37 °C for 24 h for determination of the number of viable cells. Each sample was tested in triplicate and in three independent experiments, for evaluation of biocidal performance.

### 2.8. Testing of Biocidal Stability and Rechargeability

The biocidal modifier is gradually worn away from the surface and the *N*-halamine sites are progressively hydrolyzed during usage and storage, causing the loss of antibacterial capacity. Therefore, it was important to evaluate the stability and rechargeability of the biocidal function. The stability and rechargeability biocidal polysiloxane modifier and imide *N*-halamine sites toward washing cycles were tested using a standard method of the American Association of Textile Chemists and Colorists (2A procedure of AATCC 61−1996), in which 2.5 × 5.1 cm^2^ of swatches were placed in a canister with 150 mL of 0.15% AATCC detergent and 50 stainless steel balls. The canister was rotated at 42 rpm and 49 °C for 45 min to complete one washing cycle, which was equivalent to five machine cycles. Washed swatches were subsequently cleaned with distilled water, followed by drying in the air at room temperature. Some of the dried swatches were directly titrated for assessment of the concentration of residual chlorine, while others were first rechlorinated with *tert*-butyl hypochlorite and then subjected to titration for evaluation of the rechargeability of the lost chlorines.

The stability and rechargeability of biocidal functionality for long-term storage were assessed by storing cyclic imide *N*-halamine polysiloxane@cellulose samples at 25 °C and 65% relative humidity (RH) for up to 3 months under laboratory light from fluorescent lamps. One set of stored samples was immediately titrated and the other set was rechlorinated and subsequently titrated, to evaluate the retention and regeneration of *N*-halamine structures.

The stability and rechargeability toward UV irritation (~340 nm) were investigated using an AP-UV Accelerated Weathering Tester (Dongguan Aipei Test Equipment Co., Ltd., Dongguan, China).) at room temperature and 25% RH. After a certain time of UV exposure, the swatches with and without rechlorination were subjected to iodimetric/thiosulfate titration, to estimate the photolytic stability of the polysiloxane modifier and *N*-halamine sites of cyclic imide *N*-halamine polysiloxane@cellulose.

## 3. Results and Discussion

### 3.1. Synthesis of Barbituric Acid-Based Polysiloxane

**Figure 1:**
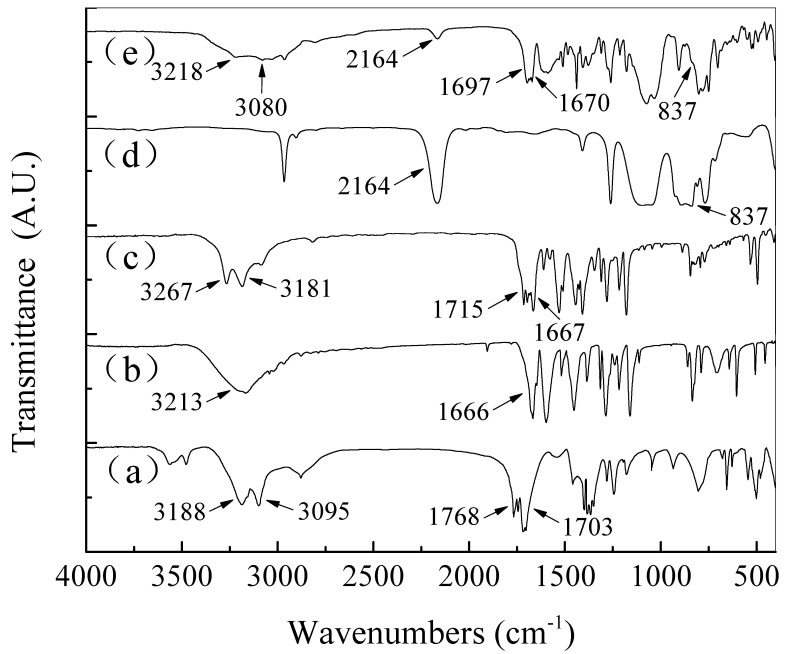
FTIR spectra of barbituric acid (**a**), 4-hydroxybenzaldehyde (**b**), 5-(4-hydroxybenzylidene)pyrimidine-2,4,6-trione (**c**), PMHS (**d**), and barbituric acid-based polysiloxane (**e**).

The synthesis of barbituric acid-based polysiloxane includes two steps, as shown in Scheme 1. The first is the preparation of 5-(4-hydroxybenzylidene)pyrimidine-2,4,6-trione via Knoevenagel condensation between barbituric acid and 4-hydroxybenzaldehyde. The FTIR spectra of barbituric acid, 4-hydroxybenzaldehyde, and 5-(4-hydroxybenzylidene)pyrimidine-2,4,6-trione are presented in Figure 1. In the spectrum of barbituric acid (Figure 1a), the characteristic peaks at 3188 and 3095 cm^−1^ are assigned to the stretching vibrations of N−H, and the ones in the range of 1703 to 1768 cm^−1^ are attributed to stretching vibrations of the three different types of C=O bonds, respectively. In the spectrum of 4-hydroxybenzaldehyde (Figure 1b), there are bands at 1666 and 3213 cm^−1^ that are attributed to stretching vibrations of C=O and phenolic O−H, respectively. The spectrum of 5-(4-hydroxybenzylidene)pyrimidine-2,4,6-trione exhibits features originating from both reactants, such as overlapped peaks of stretching vibrations of O−H and N−H at 3181 to 3267 cm^−1^ and stretching vibration of C=O bonds originating from barbituric acid in the range of 1667 to 1715 cm^−1^, respectively, as shown in Figure 1c.

In the second step, 5-(4-hydroxybenzylidene)pyrimidine-2,4,6-trione was reacted with PMHS via silane alcoholysis. In the spectrum of PMHS, the stretching and bending peaks of Si−H groups appeared at 2164 and 837 cm^−1^, respectively, as shown in Figure 1d, respectively. However, the intensities of these two peaks were greatly reduced in the spectrum of the silane alcoholysis product (Figure 1e), barbituric acid-based polysiloxane, due to the consumption of Si−H groups in the alcoholysis reaction. The peaks from 5-(4-hydroxybenzylidene)pyrimidine-2,4,6-trione, including the stretching mode of N−H and C=O, also occur in the spectrum at similar wavenumbers of 3218 and 3080 cm^−1^ and in the range of 1670 to 1697 cm^−1^, respectively. The FTIR characterization proved the successful synthesis of barbituric acid-based polysiloxane.

### 3.2. Construction and Characterizations of Biocidal Polysiloxane Layer on Cellulose

The relationship between interpenetration pressure and the thickness of barbituric acid-based polysiloxane on cellulose was first studied. The solvent strength of scCO_2_ increases with the increase of pressure, which leads to a higher solubility of barbituric acid-based polysiloxane and, therefore, a thicker modification layer on cellulose. Therefore, variation of interpenetration pressure is a convenient method to adjust the thickness/delivery ability of barbituric acid-based polysiloxane, for control of the loading of biocidal *N*-halamine, whose efficacy is content-dependent, and to achieve different killing capacities.

**Figure 2:**
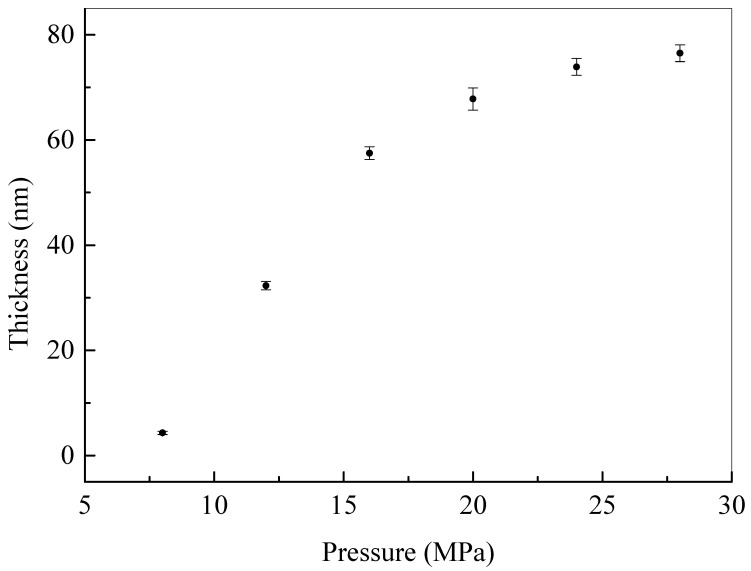
Thickness of barbituric acid-based polysiloxane layer as a function of interpenetration pressure.

The solubility of a polymer in scCO_2_ is very sensitive to pressure. The relationship of the thickness of barbituric acid-based polysiloxane layer and interpenetration pressure is presented in Figure 2. It was observed that the thickness of the modifier increased sharply in the pressure region from 8 to 20 MPa and then more gradually at higher pressures, reaching a maximum thickness of 76.5 nm at 28 MPa. Therefore, it is quite convenient to control the loading of the modifier to deliver different amounts of cyclic imide *N*-halamine onto cellulose, eventually through the adjustment of interpenetration pressure. The cellulose with the thickest modifier (76.5 nm) was chlorinated to produce cyclic imide *N*-halamine polysiloxane@cellulose, which was used for following characterizations and tests.

**Figure 3:**
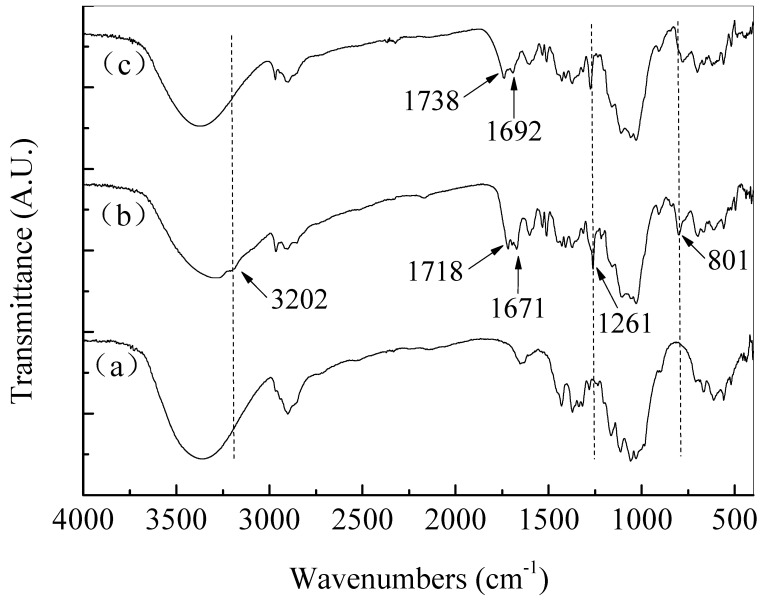
FTIR spectra of pristine cellulose (**a**), barbituric acid-based polysiloxane@cellulose (**b**), and cyclic imide *N*-halamine polysiloxane@cellulose (**c**).

The composition changes of cellulose caused by interpenetration and chlorination were first characterized using the FTIR technique. The spectra of unmodified cellulose, barbituric acid-based polysiloxane@cellulose, and cyclic imide *N*-halamine polysiloxane@cellulose are shown in Figure 3. Compared with that of pristine sample, the spectrum of cellulose interpenetrated with barbituric acid-based polysiloxane (Figure 3b) shows characteristic peaks arising from barbituric acid-based polysiloxane, including an imidic N−H stretching vibration centered at 3202 cm^−1^, C=O stretching vibrations of NHCONH and NHCOC< modes in the range of 1671 to 1718 cm^−1^, a Si–O–C stretching mode at 1261 cm^−1^, and Si−O−Si stretching mode at 801 cm^−1^. After chlorination, the stretching peak of N−H vanished due to the transformation to N−Cl, as shown in Figure 3c. In addition, the stretching vibrations of different C=O bonds appeared in a higher wavenumber range of 1692 to 1738 cm^−1^. This shift of C=O stretching peak was also been observed in other cases and is believed to be caused by the disappearance of hydrogen bonding of N–H---O=C and the increase of atomic mass when using chlorine to replace hydrogen [32,42]. Therefore, FTIR proved the success of the interpenetration and chlorination processes. The loading of the biocidal chlorine on cyclic imide *N*-halamine polysiloxane@cellulose was calculated to be 0.31 wt% based on data of titration using Equation (3).

**Figure 4:**
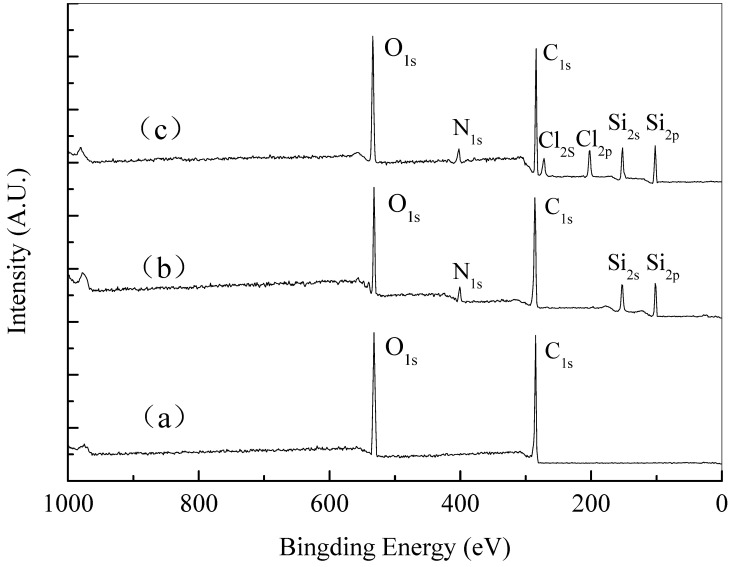
XPS wide scans of pristine cellulose (**a**), barbituric acid-based polysiloxane@cellulose (**b**), and cyclic imide *N*-halamine polysiloxane@cellulose (**c**).

**Figure 5:**
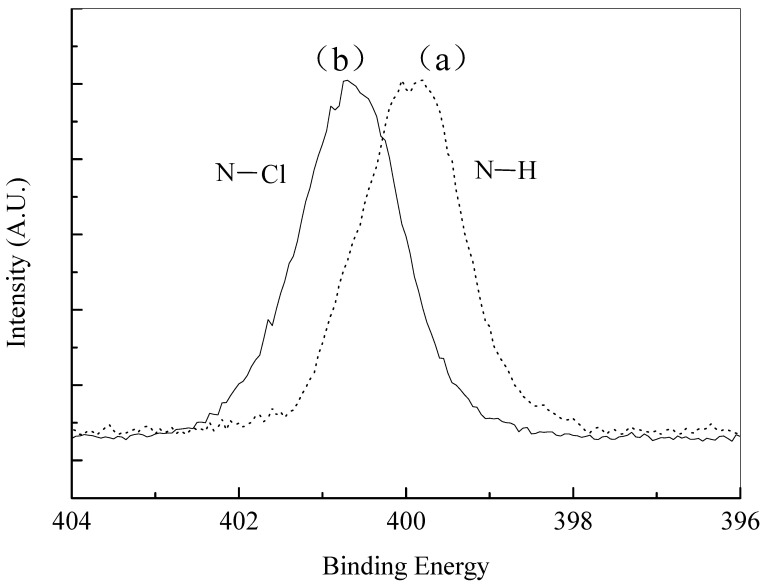
N_1s_ high-resolution scans of barbituric acid-based polysiloxane@cellulose (**a**) and cyclic imide *N*-halamine polysiloxane@cellulose (**b**).

XPS was subsequently used as a supplement to FTIR, since it merely collects information on the top surface (~5 nm), which avoids signal interference with the cellulose substrate. XPS survey scans of pristine cellulose, barbituric acid-based polysiloxane@cellulose, and cyclic imide *N*-halamine polysiloxane@cellulose are shown in Figure 4. The spectrum of pure cellulose only displays photoelectron peaks of C_1s_ and O_1s_ at approximate 285 and 532 eV, as shown in Figure 4a. The spectrum of barbituric acid-based polysiloxane@cellulose (Figure 4b) essentially shows photoelectron peaks of elements of the barbituric acid-based polysiloxane modifier, including Si_2p_, Si_2s_, C_1s_, N_1s_, and O_1s_ at about 102, 152, 285, 400, and 532 eV, respectively. The spectrum of the chlorinated sample, cyclic imide *N*-halamine polysiloxane@cellulose, exhibited two more peaks of Cl_2p_ and Cl_2s_ at around 202 and 272 eV, as illustrated in Figure 4c, respectively, owing to the transformation of N−H to N−Cl. In addition, chlorination caused a change of chemical status of nitrogen. Then, high resolution spectra of N_1s_, before and after chlorination, were acquired, as shown in Figure 5, which displays a binding energy shift from 399.9 eV to 400.7 eV, confirming the transformation of the N−H structure to its N−Cl counterpart [26,43].

**Figure 6:**
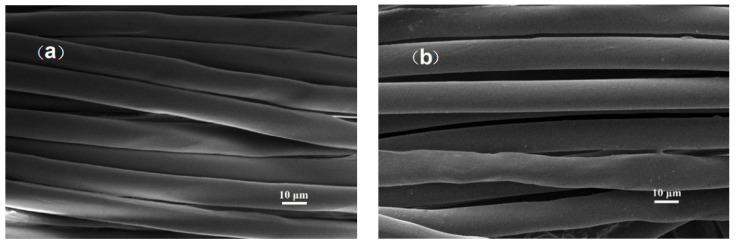
SEM images of pristine cellulose (**a**) and cyclic imide *N*-halamine polysiloxane@cellulose (**b**).

The morphology of the biocidal polysiloxane modifier on cyclic imide *N*-halamine polysiloxane@cellulose was observed using SEM, after the verification of its existence with FTIR and XPS. SEM images show that a continuous and slightly coarse film fully covered the original smooth surface of pristine cellulose, comparing Figure 6a,b. A defect-free modifier with the correct chemical composites ensured the desired biocidal performance, which was subsequently confirmed through antibacterial testing. Cellulose fibers swell in scCO_2_ but there was no measurable change in diameter after depressurization (image not shown for brevity).

### 3.3. Antibacterial Testing

The antibacterial function of cyclic imide *N*-halamine polysiloxane@cellulose was measured against the two model strains Gram-negative *E. coli* and Gram-positive *S. aureus,* using pristine cellulose as a negative control. The log reductions of the two bacteria at different contact durations with biocidal swatches and controls are summarized in Table 1.

The controls did not afford any perceptible reduction of both *E. coli* and *S. aureus,* even at 20 min of contact, since the slight decreases of *E. coli* (0.37 log) and *S. aureus* (0.36 log) were caused by natural mortality and adsorption of microbes onto the cellulose fibers. In contrast, cyclic imide *N*-halamine polysiloxane@cellulose swatches exerted a powerful biocidability, achieving 3.96, 5.22, 6.78, and 7.11 log reductions of *E. coli* and 3.42, 4.76, 6.12, and 7.08 log reductions of *S. aureus* at contact time of 5, 10, 15, and 20 min, respectively. The slightly lower efficacy against *S. aureus* could be attributed to its thicker membrane, which is more resistant to the penetration of biocidal chlorines. The efficacy herein was higher than that of amide and amine *N*-halamines at a similar chlorine loading, which needed a longer contact time (20–30 min) to kill nearly the same number of bacteria [44,45], which proved the advantage of imide *N*-halamine, which has the highest dissociation constant and the fastest release of chlorine ions, to provide the most efficient killing. In addition, each barbituric acid unit has two *N*-halamine sites and hence a higher ratio of biocidal functionality, which benefits the antibacterial performance.

### 3.4. Biocidal Stability and Rechargeability

The biocidal stability and rechargeability of cyclic imide *N*-halamine polysiloxane@cellulose are important parameters for the evaluation of the antibacterial group, polysiloxane modifier, and modification procedure. These two properties were therefore studied against repeated washings, UV irradiation, and storage. Polysiloxanes are ideal biocidal carriers, since they are sturdy, hydrophobic, and photodegradation resistant. As shown in Table 2, the loading of biocidal chlorine decreased with the increase of washing cycles, since the polysiloxane modifier was gradually washed away and the *N*-halamine sites progressively hydrolyzed, from an initial value of 0.31 wt% to 0.10 wt% and to 0.06 wt% after 5 and 10 AATCC washing cycles, respectively. The washing stability of the biocidability was very good, since even after 10 AATCC washing cycles, the chlorine loading was still higher than 0.05 wt%, the threshold value for a total kill of ~10^7^ CFU bacteria [46]. Although the lost part of the biocidability caused by leaching of the modifier cannot be regained, the rechargeability that was contributed by rechlorination of the hydrolyzed *N*-halamine sites was still very satisfactory, since the samples after 10 AATCC washing cycles could be recharged to a chlorine loading of 0.10 wt% with *tert*-butyl hypochlorite. The good washing stability and rechargeability are attributed to several factors. The first is that the polysiloxane backbone is hydrophobic and has high bond energy, so that the modifier is resistant to washing cycles. The second is that a cyclic *N*-halamine is more stable than its acyclic counterpart. In addition, this also indicates that the biocidal polysiloxane was interpenetrated to a sufficient depth into the cellulose in scCO_2_, forming a strong interlocking structure. The method of scCO_2_ interpenetration does not rely on chemistry and hence is transferable to other substrates, including PET and polyolefins.

The data of degradation and rechlorination of biocidal chlorines of cyclic imide *N*-halamine polysiloxane@cellulose under UV irritation are summarized in Table 3. The chlorine loading declined rapidly within the first two hours, losing 55% of the initial amount. Then, the decrease became slower, reaching chlorine loadings of 0.02 wt% and 0 wt% at 24 h and 7 d, respectively. This observation of a negative acceleration is believed to be due to the polysiloxane backbone on top surface that is photodegraded to inorganic SiO_x_, which protected the components beneath from rapid decomposition [47]. The partial photodegradation of the polysiloxane modifier is the reason why the biocidability of the irradiated samples cannot be fully regained. The rechargeability of lost chlorines due to UV exposure was promising, since chlorine loadings of 0.19 wt% and 0.17 wt% for the samples irradiated for 24 h and 7 d were obtained, respectively.

The imide *N*-halamine sites underwent hydrolysis to furnish N−H bonds during storage, due to the adsorption of moisture from air. The formed N−H bonds can be rechlorinated to recover the biocidability. However, the hydrolysis of an *N*-halamine produces an HOCl molecule that can trigger acid-catalyzed decomposition or oxidation of components of the biocidal layer, leading to a permanent loss of chlorine. Since both washing and storage involve hydrolysis of *N*-halamine sites, it is naturally expected that the washing-resistant biocidability described above can be also maintained during long-term storage. The test results proved this expectation: the loading of chlorine on cyclic imide *N*-halamine polysiloxane@cellulose was merely reduced by 9.0%, from 0.31 wt% for fresh samples to 0.28 wt% for the samples stored at 25 °C and 65% RH in an air-conditioned chamber for 3 months. Almost all of the lost chlorines in this study could be recharged, which indicates that the loss of chlorines was due to the hydrolysis of N−Cl to N−H instead of irreversible chemical changes. This good storage stability and rechargeability are attributed to the hydrophobic delivery vehicle of polysiloxane, which does not adsorb moisture rapidly, and the cyclic *N*-halamine structure, which is more stable than an acyclic one. However, more than 85% of acyclic *N*-halamine sites were lost in only 1 month at the same temperature and RH, when using hydrophilic chitosan as a carrier that quickly adsorbs a large quantity of moisture, to facilitate the hydrolysis of less stable, open-chain N−Cl bonds [19]. Therefore, the biocidal chlorines of cyclic imide *N*-halamine polysiloxane@cellulose can be preserved for a long period, to offer sufficient antibacterial ability. The biocidal modifier was relatively thin in this study, due to the low solubility of barbituric acid-based polysiloxane in scCO_2_, which only has a poor solvent strength. A thicker modifier is desirable, since it is more damage-resistant and ensures a more durable biocidability. An increase of the interpenetration pressure can partially resolve this problem.

## 4. Conclusions

A polysiloxane delivery vehicle was designed to carry cyclic imide *N*-halamine sites onto cellulose using scCO_2_ interpenetration, to offer a powerful, durable, and rechargeable biocidability. For this purpose, a barbituric acid-based polysiloxane was first synthesized using a two-step reaction between barbituric acid and 4-hydroxybenzaldehyde, followed by silane alcoholysis of the resultant sample with of PMHS. The barbituric acid-based polysiloxane was interpenetrated into cellulose in scCO_2_ at 50 °C and formed a layer whose thickness increased with the increase of pressure, with a maximum value of 76.5 nm at 28 MPa, showing the ability for adjustable delivery of the control of the loadings of imide N−H and therefore N−Cl. After chlorination of cyclic imide N−H bonds of barbituric acid pendants to *N*-halamine formats, the final cyclic imide *N*-halamine polysiloxane@cellulose provided powerful biocidability, killing *S. aureus* and *E. coli* with populations of ~10^7^ CFU in 20 min. The biocidal stability and rechargeability towards washing, UV irradiation, and storage were also promising compared with previously reported cases. These features of biocidability are attributed to the sturdy, photo-resistant, and hydrophobic polysiloxane backbone and to the stable and efficient cyclic imide *N*-halamine. This study also showed that scCO_2_ interpenetration is a benign and general surface modification method, since it does not use a polluting solvent or rely on the chemistry of the substrate. The produced antibacterial cellulose has broad applications in both daily life and the healthcare field, due to its ability to prevent bacterial infection.

## Figures and Tables

**Table 1:** Antibacterial function of cyclic imide *N*-halamine polysiloxane@cellulose and pristine control.

Material	Contact Time (min)	Log Reduction of *E. coli*	Log Reduction of *S. aureus*
Pristine control	5	0.13 ± 0.07	0.14 ± 0.08
	10	0.18 ± 0.08	0.19 ± 0.06
	15	0.25 ± 0.06	0.27 ± 0.06
	20	0.37 ± 0.05	0.36 ± 0.06
Cyclic imide *N*-halamine polysiloxane@cellulose	5	3.96 ± 0.07	3.42 ± 0.06
	10	5.22 ± 0.07	4.76 ± 0.07
	15	6.78 ± 0.06	6.12 ± 0.06
	20	7.11 ± 0.00	7.08 ± 0.00

*S. aureus* and *E. coli* at an inoculum population of 1.2 × 10^7^ and 1.3 × 10^7^ CFU, respectively.

**Table 2:** Washing stability and rechargeability of the biocidability of cyclic imide *N*-halamine polysiloxane@cellulose.

No. of Washing Cycles	Remained Chlorine (wt% Cl^+^)	Recovered Chlorine after Rechlorination (wt% Cl^+^)
1	0.18 ± 0.01	0.24 ± 0.02
3	0.13 ± 0.02	0.17 ± 0.01
5	0.10 ± 0.01	0.15 ± 0.02
10	0.06 ± 0.01	0.10 ± 0.01

**Table 3:** Biocidal stability toward UV irradiation of the cyclic imide *N*-halamine polysiloxane@cellulose.

Exposure Time	1 h	2 h	4 h	8 h	12 h	24 h	7 d
Remained chlorine (wt%)	0.18 ± 0.01	0.14 ± 0.02	0.09 ± 0.01	0.06 ± 0.01	0.03 ± 0.01	0.02 ± 0.01	0 ± 0.00
Recovered chlorine (wt%)	0.26 ± 0.02	0.24 ± 0.01	0.23 ± 0.02	0.21 ± 0.01	0.20 ± 0.01	0.19 ± 0.01	0.17 ± 0.01

## Data Availability

The data presented in this study are available on request from the corresponding author.
